# A C-banded karyotype of mitotic chromosomes in diploid purple coneflower (*Echinacea purpurea* L.)

**DOI:** 10.1186/s41065-016-0016-y

**Published:** 2016-11-22

**Authors:** Weizhen Jiang, Qingling Li, Xiaolu Chen, Yi Ren, Rong Chen, Hong Wu, Yuesheng Yang

**Affiliations:** 1 Genetic Engineering Laboratory, College of Life Sciences, South China Agricultural University, Room 614, Guangzhou, 510642 People’s Republic of China; 2 Research Center of South China Medicinal Plants, South China Agricultural University, Guangzhou, 510642 People’s Republic of China; 3 Guangdong Technology Research Center for Traditional Chinese Veterinary Medicine and Natural Medicine, South China Agricultural University, Guangzhou, 510642 People’s Republic of China

**Keywords:** Karyotype, C-banding technique, Aneuploid, *E. Purpurea*, Chromosomes

## Abstract

**Background:**

Aneuploid ermpglasm is an important resource for genetic studies and identification of individual chromosomes in the cells of the aneuploid is an important step. The karyotype has already been established for purple coneflower (*Echinacea purpurea* L.), but due to the high similarity in the morphology of several pairs of chromosomes in this species, it cannot be used to identify individual chromosomes in its own complement. The objectives of this study are to develop and evaluate the Giemsa C-banding technique for the purpose of identifying the individual chromosomes in *Echinacea purpurea*.

**Results:**

The established karyotype with C-bands showed that all the 11 pairs of chromosomes possessed centromeric bands. Telomeric bands appeared most frequently in almost all the chromosomes with only two exceptions, the short arm of the chromosome 9 and the long arm of the chromosome 10. Intercalary bands were found mainly in the long arm of some chromosomes with only two exceptions, the chromosomes 1 and 2 that had intercalary bands on both arms. The chromosome 4 was the only chromosome where intercalary bands were absent.

**Conclusions:**

Chromosomes in *E. purpurea* could be stained with Giemsa to bear C-bands. By classifying the chromosomes into groups and judging the C-bands, each chromosome could be identified. The methods established in this study might be used for the identification of chromosome constitution in aneuploid *E. purpurea* created in a breeding program.

## Background

Purple coneflower (*Echinacea purpurea* L.) is native to North America [1, 2] and has important pharmaceutical [3, 4] and ornamental [5] values. Wild *E. purpurea* plants are diploids with 22 chromosomes in somatic cells [6, 7]. The karyotypes of *Echinacea* species have been reported to be quite similar [6, 8]. In 2004, the karyotype of *E. purpurea* was first established by Qu et al.; it has been used successfully to differentiate *E. Purpurea* from another *Echinacea* species, *E. angustifoli*, based on the difference in the centromere’s position of chromosome pair 10 between the two species. However, due to the high similarity in the morphology of some pairs of the chromosomes in *E. purpurea*, the karyotype established by Qu et al. [8] cannot be used to identify every chromosome in the species.

Ploidy breeding which includes polyploids [9, 10] and aneuploids [11, 12] has been proved to be a feasible method for many plant species. Following the success in obtaining tetraploid *E. purpurea* by in vitro colchicine treatment of diploid explants (Nilanthi et al. [13], triploid plants were obtained by conventional crossing method between diploid and tetraploid plants [14] while aneuploids were obtained by crossing between diploid and triploid plants (unpublished results). To obtain aneuploid in *E. purpurea* seems easy because aneuploid individuals are known to exist naturally in Asteraceae [15, 16], the family to which *E. purpurea* belongs. Aneuploids are different from euploids based on the possession of more or less number of chromosomes and are a unique germplasm resource for genetic studies [17–19]. Before an effective study can take place, clarification of the constitution of the chromosomes in the material aneuploid is a prerequisite.

Similarity in morphology among some pairs of chromosomes in cells seems a common feature in many plant species. In order to distinguish individual chromosomes from each other in the complement, many chromosome banding techniques such as C-banding [20], G-banding [21], N-banding [22], Q-banding [20, 23] and R-banding [24] have been attempted. Among these banding techniques, C-banding staining of the constitutive heterochromatin of the chromosomes with Giemsa is the most frequently used one in a range of plant species [25–28]. In this report, we describe an effective karyotype based on chromosome length, arm ratio, and the C-banding patterns for identifying individual chromosomes in *E. purpurea*.

## Method

Seeds of purple coneflower (*Echinacea purpurea* L.) were originally purchased at a supermarket provided by the Company of Plantation Products (Norton, MA 02766, USA) and maintained by harvesting seeds from the plants cultivated in the campus of South China Agricultural University. The seeds were stored in a refrigerator at 6 °C after harvest. Before germination, the seeds were treated with 2 mg/l gibberellin acid (GA_3_) for 24 h for the purpose of breaking the dormancy.

Seedlings with 2 tiny real leaves which developed from the seeds sown in sandy soil were collected at noon. Root tips of about 10 mm were dissected after washing with pure water. The root tips were kept in a glass bottle filled with 0.05 % (w/v) colchicine water solution for 3–4 h at 4 °C to *E. purpurea* plants accumulate metaphase chromosomes in the cells, washed with running tap water for about 15 min, dipped in pure water for 8 min, and then put in Carnoy’s solution containing acetic acid and 95 % ethanol in a ratio of 1:3 (v/v) for at least 24 h at 4 °C for fixation. The fixed root tips were then washed again with running tap water for about 15 min, dipped in pure water for 8 min, and hydrolyzed in 1 *M* HCl for 5–6 min at 60 °C. After hydrolysis, the root tips were washed again with running tap water for 15 min and dipped in pure water for 8 min. Subsequently, these root tips were stained with 20 % (v/v) carbolfuchsin solution for 1–2 min, squashed on slides under a cover glass and observed under a microscope for the selection of images of well spread metaphase chromosomes.

The selected slides with well spread metaphase chromosomes were transferred to a freezer at 20 °C for 1 day and then the cover glass was removed. Immediately, the slides were dropped into 95 % (v/v) ethanol for 30 min to discolor the stain made by the carbolfuchsin solution. After discoloration, the slides were transferred into absolute ethanol twice, first for 30 min and then overnight for dehydration. Subsequently, the slides were put in an oven at 37 °C for 1 h, after which they were kept in a room and were ready for C-banding with Giemsa.

The C-banding method adopted was that provided by Tuna et al. [27] with minor modifications. Dehydrated slides were placed in 0.2 *M* HCl water solution and incubated in water bath at 60 °C for 2, 4, 6, 8 and 10 min, after which they were immediately washed with pure water. The slides were put into freshly prepared saturated Ba(OH)_2_ solution at room temperature for 4, 8, 12, 16, and 20 min and washed carefully in distilled water kept at 45 °C. The water was changed three times at 2 min intervals. Then, the slides were washed three times with pure water of normal room temperature to ensure that all the barium crystals were removed. After the above treatments, the slides were dried in an oven at 37 °C for about 30–45 min and incubated in freshly prepared 2 × SSC solution (1× is 0.15 *M* NaCl plus 0.015 *M* citric acid) (pH 7.0) kept in water bath at 60 °C for 0.5, 1.0, 1.5 and 2.0 h. After the incubation, the slides were washed with pure water very carefully this time because the chromosomes adhering to the slide glass might drop off. The slides were dried again in an oven for 30–45 min at 37 °C and stained with 10 % (v/v) Giemsa in phosphate buffer (consisting of 62 % 0.07 *M* Na_2_HPO_4_ and 38 % 0.07 *M* KH_2_PO_4_) for 10, 20, 30, 40, 50 and 60 min. After the staining, the slides were quickly rinsed with distilled water and dried for several hours at room temperature. For observations, slides were mounted in Permount.

Images of well-spread chromosomes were screened under a microscope (LeicaDLMB2) with 1000 times magnification and the photographs were taken. Eight best images with all the 11 pairs of chromosomes in one cell were selected, and the karyotype was analyzed with the software Adobe Photoshop CS5. Data for the long arm and short arm of each chromosome were statistically analyzed with the software SPSS19.0 (IBM), and significant differences were determined by Duncan’s test at 95 % level. The chromosomes were identified on the basis of their total length, arm length ratio (long/short arm), and C-banding patterns [27]. Chromosomes in the karyotype were arranged in the order of decreasing mean chromosome length [29].

## Results and discussion

In the experiments of C-banding with Giemsa for comparing various treating parameters, the best result was obtained by directly incubating the dried slides in a saturated Ba(OH)_2_ solution for 8–12 min, followed by incubation in 2 × SSC solution at 60 °C for 1.5 h and staining with 10 % Giemsa solution for 20–30 min. The step before Ba(OH)_2_ incubation treatment of the dried slides with 0.2 *M* HCl at 60 °C for 3 min, as applied in other plant species [27, 30, 31], yielded negative results and was omitted.

The swelling of the chromosomes on the slides is most likely to occur during the Giemsa staining course. On the swollen chromosomes, only the centromeric region could be stained; the telomeric region could seldom be stained while the intercalary region could never be stained. Treatment of Ba(OH)_2_ solution for longer than 15 min or at a pH value of 2 × SSC higher than 8.0, was the main cause of the swelling of the chromosomes, leading to very poor staining results. The pH value of the phosphate buffer for preparing Giemsa solution is another important factor that needs to be mentioned. A pH value in the range of 6.8–7.0 was desirable. Higher or lower values could result in complete failure.

**Fig. 1:**
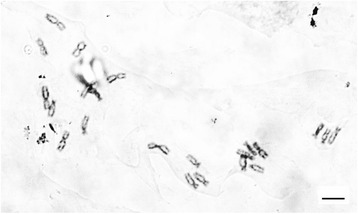
C-banded mitotic metaphase chromosomes of *E. Purpurea* (2n = 22). Bar = 10 μm

**Fig. 2:**
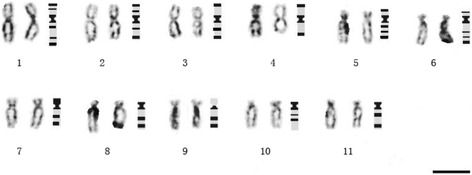
C-banded karyotype of diploid *E. purpurea* based on the cell presented in Fig. 1. Bar =10 μm

C-banded mitotic metaphase chromosomes of diploid *E. Purpurea* are shown in Fig. 1 and a detailed C-banded karyotype is presented in Fig. 2, with the accompanying total lengths and arm length ratios in Table 1. Standard deviations were also calculated for each chromosome (Table 1). The relatively small deviation values in arm lengths and arm ratios show the high quality of preparation of the chromosomes in this study.

**Table 1:** The chromosomes of the diploid *E. Purpurea* based on eight cells^a^

Code of chromosome	Length (μm) of long arm	Length (μm) of short arm	Total length (μm)	Arm ratio^b^	Type of chromosome^c^
I	6.54 ± 0.14	4.71 ± 0.18	11.25 ± 0.32	1.40 ± 0.02	m
II	5.67 ± 0.53	3.80 ± 0.21	9.46 ± 0.73	1.50 ± 0.05	m
III	5.25 ± 0.22	4.22 ± 0.13	9.46 ± 0.35	1.25 ± 0.02	m
IV	4.98 ± 0.47	3.76 ± 0.35	8.74 ± 0.81	1.34 ± 0.01	m
V	6.01 ± 0.13	2.56 ± 0.05	8.59 ± 0.15	2.40 ± 0.02	sm
VI	6.52 ± 0.91	1.73 ± 0.18	8.25 ± 1.09	3.78 ± 0.11	st
VII	5.61 ± 0.18	2.16 ± 0.15	7.76 ± 0.32	2.62 ± 0.08	sm
VIII	5.99 ± 0.14	1.75 ± 0.03	7.74 ± 0.11	3.47 ± 0.15	st
IX	6.03 ± 0.70	1.49 ± 0.16	7.55 ± 0.89	4.17 ± 0.08	st
X	5.79 ± 0.70	1.52 ± 0.21	7.30 ± 0.90	3.93 ± 0.01	st
XI	5.73 ± 0.20	1.48 ± 0.04	7.20 ± 0.23	3.99 ± 0.03	st

^a^Data were mean ± standard deviation

^b^Armratio: Length of the long arm/length of the short arm

^c^m: Arm ratio lower than 1.70; sm: arm ratio higher than 1.70 but lower than 3.0; st: arm ratio higher than 3.00 [29]

The mean lengths of the chromosomes ranged from 11.25 to 7.20 μm while the mean arm ratios ranged from 1.25 to 4.17. Arm ratios of chromosomes have been suggested by Li and Chen [29] as the only factor for classifying chromosome types in standardized karyotype analysis. In accordance with their method, within the chromosome complement in *E. purpurea*, four pairs of chromosomes were classified as metacentric (m) (chromosomes 1, 2, 3, and 4, coincides with the longest four chromosomes), two pairs were classified as submetacentric (sm) (chromosomes 5 and 7) while the remaining 5 pairs were classified as subterminal (st) (Table 1).

With proper Giemsa staining method, all the chromosomes showed terminal bands with only two exceptions, the short arm of the chromosome 9 and the long arm of the chromosome 10. Intercalary bands were observed mainly on the long arms with only two exceptions, the chromosome 1 and 2 that had intercalary bands on both arms. The chromosome 4 was the only chromosome where intercalary bands were absent.

Differentiating each chromosome among the complement in *E. purpurea* was attempted within each group of m, sm and st because the minimal differences in arm ratio between m and sm types of chromosomes and between sm and st types of chromosomes were large and could be easily observed in the present case (the maximal arm ratio for m is 1.50 while the minimal arm ratio for sm is 2.40, equal to a difference of 37.5 %; the maximal arm ratio for sm is 2.62 while the minimal arm ratio for st is 3.47, equal to a difference of 24.5 %). The first group is type m chromosomes consisting of 1, 2, 3 and 4 chromosomes. Among the four chromosomes, 1 is first identified from the others because 1 is the only one having two intercalary bands on the long arm; 2 can be identified from the others because it has one intercalary on both short arm and long arm; 3 can be identified from 4 because it has an intercalary band on the long arm.

The second group is type sm chromosomes consisting of only two chromosomes 5 and 7. They are very similar in total length and arm ratio (similarity 90.3 and 91.6 %, respectively), and both possess two telomeric bands, one centromeric band and several intercalary bands. The only way to differentiate them from each other is by comparing the number of the intercalary band: clearly, chromosome 5 has two intercalary bands on the long arm, but chromosome 7 has only one.

The third group is type st chromosomes consisting of five chromosomes 6, 8, 9, 10 and 11. They are quite similar in total length and arm ratio (maximal similarity 98.6 and 98.5 % respectively), but each of them has different patterns of C-bands and can be easily differentiated from each other. Chromosome 6 has telomeric bands and centromeric bands on both arms, different from the other four chromosomes that have two intercalary bands on the long arm. Chromosome 8 is extremely similar to chromosome 11 in length and arm ratio, and in addition both of them possess two bands on the long arm. However, the two bands on chromosome 11 are more separated. Chromosome 9 is the only chromosome without C-bands on the short arm, while chromosome 10 is the only chromosome without telomeric bands on the long arm.

The C-banded karyotype shown in Fig. 2 does not only tell the differences among the 11 pairs of chromosomes, but it also reveals certain heterogeneous characteristics between most of the homologous chromosomes. For example, in the pair of chromosomes 1, the centromeric band and an intercalary band on the long arm near the centromere were clearly different in the intensity of staining. Similar phenomena were also observed on chromosomes 2, 3, 4 and 10. The heterogeneous characteristics in C-bands regarding differences in depth of staining and banding positions between homologous chromosomes have been reported in many cases with explanations such as translocation between homologous chromosomes and differences in the content of DNA repeats in constitutive heterochromatin [32–34]. Regarding the studied plant species, *E. purpurea* is a highly self-incompatible plant [35, 36] pollinated with pollen grains mainly from other genotype plant individuals. For this, the homologous chromosomes are almost of different plant origins and might differ to a certain degree in such as DNA sequence and even chromosome configuration, causing the differences in the banding patterns in some pairs of chromosomes.

## Conclusion

In conclusion, chromosomes in *E. purpurea* could be stained to show bands. By classifying the chromosomes into groups and judging the C-bands, each chromosome could be identified. The methods established in this study might be used for the identification of chromosome constitution in aneuploid *E. purpurea* created by crosses between diploid and triploid plants.
